# A scoping review of clusters of multiple long-term conditions in people with intellectual disabilities and factors impacting on outcomes for this patient group

**DOI:** 10.1177/17446295221107275

**Published:** 2022-06-13

**Authors:** Claire Mann, Gyuchan T Jun, Freya Tyrer, Reza Kiani, Gemma Lewin, Satheesh K Gangadharan

**Affiliations:** School of Design and Creative Arts, 5156 Loughborough University, Loughborough, UK; Biostatistics Research Group, Department of Health Sciences, 4488 University of Leicester, Leicester, UK; 9632 Leicestershire Partnership NHS Trust, Leicester, UK

**Keywords:** intellectual disabilities, learning disabilities, multiple long-term conditions, scoping review

## Abstract

People with intellectual disabilities (ID) are vulnerable to multiple long-term conditions (MLTC). However, in the UK, there are no individual strategies tailored for them. This study synthesised evidence on prevalence of MLTC in people with ID alongside risk factors, outcomes and preventative strategies. The scoping review used the tool Abstrackr to search retrieved articles from three bibliographic databases. Of 933 articles initially screened and further identified, 20 papers met our inclusion criteria. Our findings revealed significant data on prevalence of MLTC in people with ID across the studies, but very limited data on clusters or patterns of co-occurrence in this population. The majority of papers explored risk factors and strategies for prevention of MLTC, but far fewer compared outcomes by MLTC. The identified gaps in the literature indicate the need for further research to identify clusters of MLTC and tailored prevention strategies to reduce poor outcomes in this population.

## Introduction

Multiple long-term health conditions (MLTC), defined here as two or more long-term health conditions (e.g.: epilepsy and dementia or epilepsy, visual impairment and diabetes), place considerable burden on health and social care settings and are at the forefront of policy initiatives around the world (Afshar et al., 2015; Chandraratne et al., 2018; Garin et al., 2016; MacMahon et al., 2018; National Institute for Health Research (NIHR), 2020; Rijken et al., 2017). People with intellectual disabilities are particularly vulnerable to MLTC in addition to their intellectual disability owing to a combination of genetic, behavioural and social factors (Cooper et al., 2015; Emerson and Hatton, 2014). Evidence points to a much higher prevalence of MLTC in this population with one large population-based study showing the mean number of conditions as high as 11 and a prevalence of MLTC (≥2 conditions in additional to intellectual disability) of 98.7% (Kinnear et al., 2018). Increased prevalence of MLTC in this population contributes to premature mortality and also affects quality of life (Heslop and Hoghton, 2018). This is a population already facing significant discrimination and barriers to accessing healthcare (Ali et al., 2013). Despite this, there appears to be little research on patterns of MLTC in this population which has led to calls for more research in this area (Kinnear et al., 2018).

In the UK, there are no individual strategies tailored for people with intellectual disabilities who also have MLTC. Intellectual disabilities were initially not included in the guidance published by the National Institute for Health and Care Excellence (NICE) on MLTC (Heslop, 2014) and now only form part of the definition of MLTC (NICE, 2016). More recent policies allude to intellectual disabilities (but do not specifically name them) in the context of “complex care needs” requiring access to numerous health, education and social care services (NIHR, 2020). Elsewhere, intellectual disabilities are not named in the definition of MLTC (Le Reste et al., 2013; World Health Organisation, 2016) but are sometimes referred to under the umbrella term “developmental disabilities” (US Department of Health and Human Services, 2010). Lack of consideration of people with intellectual disabilities in MLTC policy initiatives risks overshadowing the complexity of health needs that they experience, in particular consideration of reasonable adjustments and mental capacity issues, the practice of which are often below the expected standards in this population (Heslop et al., 2013).

With this in mind, this scoping review aimed to synthesise the evidence in relation to MLTC in people with intellectual disabilities and to put forward recommendations on future work to identify groups of individuals that may benefit from focussed prevention strategies. The review formed part of a broader programme of work which sought to identify MLTC and improve outcomes and quality of life for people with intellectual disabilities (the DECODE study; see funding information) and was carried out to inform our strategy alongside views from key partners including people with ID and their carers. The overall aim was to identify relevant literature on intellectual disabilities and MLTC using the objectives below:

• Determine the prevalence of MLTC (using the definition of two or more health conditions utilised by NIHR, 2020) in adults with intellectual disabilities.
• Identify the nature of MLTC in adults with intellectual disabilities to assess whether some were more likely to occur together.
• Identify risk factors for MLTC in adults with intellectual disabilities.
• Identify outcome measures that could be used to evaluate the impact of MLTC in adults with intellectual disabilities (e.g. mortality).

We aimed to identify the evidence around these key issues to contribute to the improvement of care for people with intellectual disabilities with particular focus on the management of their MLTC.

## Methods

### Search strategy

**Figure 1.**
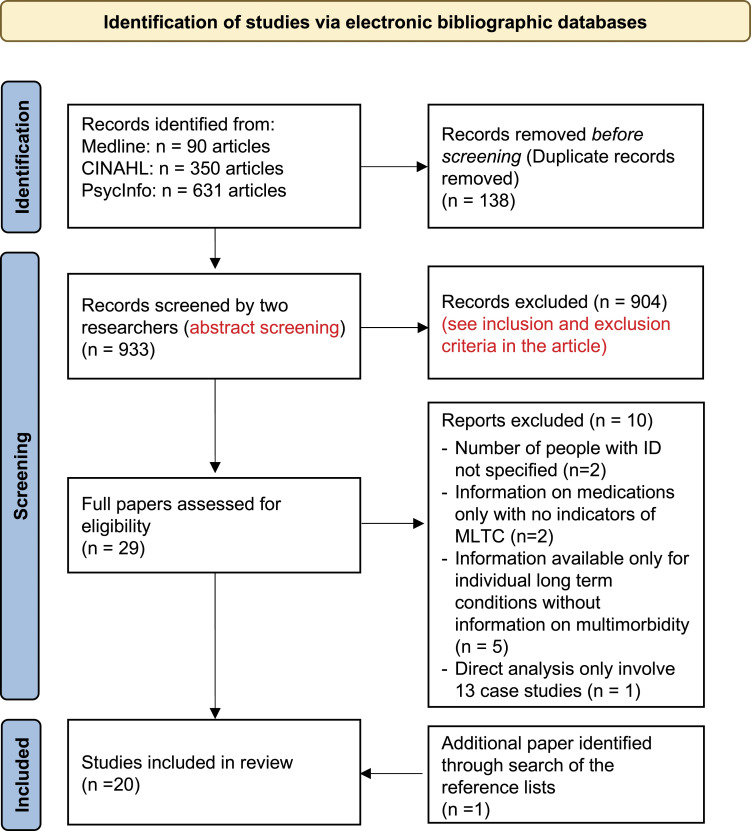
PRISMA flow diagram.

The scoping review was conducted in December 2020 using the machine learning tool Abstrackr. Three databases were searched: Medline (OVID platform); CINAHL (EBSCO platform) and PsychInfo (EBSCO platform). The search strategy for these databases is shown below (see Figure 1) and is based on previous literature (Bricca et al., 2020; Ingram et al., 2021; Soley-Bori et al., 2021; Tyrer et al., 2021). The search was limited to English language literature published from 1 January 2010, in order to gain contemporary insights into MLTC among people with intellectual disabilities over the last 10 years. Table 1 outlines the specific search strategy utilised.

**Table 1.** Search strategy.

Database	Search strategy
Medline	1. exp intellectual disability/
	2. (“learning dis*” or “developmental dis*” OR “intellectual dis*” OR “mental ret*” OR “developmental dis*” OR “down synd*”).tw
	3. 1 OR 2
	4. exp multimorbidity/
	5. (multimorbid* OR multi-morbid* OR “multiple morbidity” OR “intercurrent morbidity” OR “coexisting condition*” OR “coexisting diagnos*”).mp
	6. (“coexisting disease*” OR “coexisting illness*” OR “concurrent condition*” OR “concurrent diagnos*” OR “concurrent disease*”).mp
	7. (“concurrent illness*” OR “multiple chronic disease*” OR “several chronic diseases*” OR “multiple chronic condition*”).mp
	8. (“multiple chronic medical conditions” OR “several chronic condition*” OR “multiple health problem*” OR “multiple long-term cond*” OR “several health problems*” OR “one or more chronic conditions”).mp
	9. (“multiple illness*” OR “multiple diagnos*” OR “multiple disease*” OR “multiple pathology*” OR “multiple condition*”).mp
	10. 4 OR 5 OR 6 OR 7 OR 8 OR 9
	11. 3 AND 10
	12. Limit 11 to English language
	13. exp animals/not humans.sh
	14. 12 not 13
	15. Limit 14 to yr = “2010-Current”
CINAHL and PsycInfo	(MH Intellectual disability+ OR (“learning disabil*” OR “intellectual disabil*” OR “mental ret*” OR “developmental disabil*” OR “Down syn*” OR “mental handicap”)) AND (MH multimorbidity + OR (multimorbid* OR multi-morbid* OR “multiple morbidity” OR “intercurrent morbidity” OR “coexisting condition*” OR “coexisting diagnos*” OR “coexisting disease*” OR “coexisting illness*” OR “concurrent condition*" OR “concurrent diagnos*" OR “concurrent disease*" OR “concurrent illness*” OR “multiple chronic disease*” OR “several chronic disease*” OR “multiple chronic condition*” OR “multiple chronic medical conditions” OR “several chronic condition*” OR “multiple health problem*" OR “multiple long-term cond*” OR “several health problem*” OR “one or more chronic conditions” OR “multiple illness*” OR “multiple diagnos*” OR “multiple disease*” OR “multiple pathology*” OR “multiple condition*”))

### Inclusion and exclusion criteria

We chose a broad set of inclusion criteria to meet the range of objectives identified for this study. The inclusion criteria were primary research published in the English language reporting on adults and reporting information about two or more long-term health conditions in addition to intellectual disability. Exclusion criteria comprised animal studies, studies containing children only, clinical trials, review articles, genetic or diagnostic studies, case studies, letters and book chapters that did not report primary research.

### Screening process

All titles, abstracts and key words of the retrieved articles were uploaded to the machine learning software, Abstrackr (Wallace et al., 2012). The system has a machine learning algorithm running in the background which uses information about user choices to predict which of the abstracts yet to be screened are most likely to be relevant. The subsequent abstracts are then ordered such that articles with higher probabilities are screened first. This is designed to facilitate the screening process as those articles screened first are more likely to be relevant and less likely to be erroneously missed. Two researchers (SKG, RK) screened each article separately and any conflicts were discussed and resolved.

Figure 1 shows the PRISMA flow diagram of the 933 articles originally identified in the search, and how this reduced down to 29 articles using the inclusion/exclusion criteria described above. The 29 full articles were reviewed again (secondary review) and a further 10 were rejected. An additional article was retrieved after searching the reference lists of the papers, leaving 20 papers in total.

### Secondary review

The secondary review was undertaken by two researchers (CM, SKG) who reviewed the full text versions of each of the 29 selected articles and complete a standardised data extraction table (Cronin et al., 2008). At this stage a further 10 papers were rejected either on the basis of inclusion and exclusion criteria or ability to answer the research questions, with the addition of a further paper which was identified in a review of references resulting in a total of 20 papers included in the scoping review.

## Results

Twenty articles met the criteria for inclusion in this review from 2010 to 2021. Studies were generally conducted in high-income countries: most (*n* = 13; 65%) were from Europe (UK [*n* = 5]; Ireland [*n* = 2]; Spain [*n* = 2]; Netherlands [*n* = 3]; Italy [*n* = 1]). The remaining studies were from North America (USA [*n* = 4]; Canada [*n* = 2]) or Oceania (Australia [*n* = 1]). Included articles are summarised in Table 2, including characteristics of the study populations, type/genetic cause of intellectual disability, setting and study design.

**Table 2.** Summary of articles included in the scope of the literature.

Paper no	Author/s and date	Country of origin	(*n*)	Study population (inclusion criteria)	Study type	Setting	Prevalence of MLTC	Nature of MLTC combinations	Risk factors for MLTC	Outcome of MLTC
1	Carfi et al. (2020)	Italy	421	Adults (down syndrome) Mean age: 38 (range not available)	Cohort study: Down syndrome only. Used a standardized clinical protocol including physical exam, EKG, blood sampling, nutrition assessment.	Hospital - outpatients	88%	Not available	Older age	Not available
				Male: 49% Female: 51%						
				Ethnicity: Not reported						
2	Darbà (2019)	Spain	289	Children and adults (diagnosis of PKU) Mean age 14.9 (0–86)	Cohort study: PKU only. Used a database to identify patients with PKU in the region and data extracted from existing health records.	All	50%	Not available	Not available	Not available
				Male: 48% Female: 52%						
				Ethnicity: Not reported						
3	Jones et al. (2016)	USA	92	All adults (autism). Median age 36 (23–59)	Cohort study: people with autism. Participants identified during an epidemiological survey were recontacted.	Autism clinic (Utah)	54%	Not available	Female obesity	Not available
				Males: 75% Females: 25%						
				Ethnicity: Not reported						
4	Malecki et al. (2020)	Canada	264	Adults (diagnosis of 22q11.2DS), Mean age: 27.8 (17–68)	Cohort study: 22q11.2DS only. Data extracted from health records.	Hospital outpatients	35%	Not available	Older age psychotic illness	Not available
				Males: 47% Females: 53%						
				Ethnicity: Not reported						
5	Hodapp et al. (2019)	USA	43	Adults (downs syndrome) Age range 50–70.	Cross sectional study: Down syndrome only. Only two databases (NCI and DS connect used for identification of MLTC.	Database information (NCI, DS-Connect)	Not available	Most prevalent second conditions in those with dementia described	Not available	Not available
				Gender: Not reported						
				Ethnicity: Not reported						
6	Belton et al. (2018)	Ireland	721	Adults (Intellectual disability) Mean age 50.1 (SD6.5)	Cohort study: specialist services. Data from Irish longitudinal study on ageing. Participants identified from Irish National Intellectual disabilities database (NIDD)	All	65% (among people with down syndrome (*n* = 144). Prevalence not described in the wider group).	Not available	Older age, obesity, high medication use	Not available
				Male: 45% Female: 55%						
				Ethnicity: Not reported						
7	Tyrer et al. (2019)	UK	920	Adults (intellectual disability) Mean age 42.9 (18–74)	Cross-sectional study: people with out diabetes identified during the STOP diabetes study.	Database	61%	MLTC pairs described	Female severe/profound ID sedentary behaviour	Not available
				Males: 58% Females: 42%						
				White: 81% S. Asian: 15% Black: 1% Mixed: 1% Other: 1%						
8	Schoufour et al. (2018)	Netherlands	1050	Adults (aged 50 or over and receiving formal care three Dutch care organisations. Mean age 62 (50–93)	Cohort study: Population-based.	Dutch care settings specialised in intellectual disability care	47% (4 or more conditions). 35% (5 or more chronic medication)	Not available	Not available	Mortality
				Males: 51% Females: 49%						
				Ethnicity: Not reported						
9	Espadas et al. (2020)	Spain	83	Patients with ASD and intellectual disabilities living in residential facilities	Cohort study: autism only. Observational and multicentric pharmacovigilance study in subjects with ASD and intellectual disability.	Residential community	37% (2 or more long term conditions) 57% (5 or more long term medication)	Pairs of co-morbid conditions described.	Not available	Not available
				Males: 86% Females: 14%						
				Ethnicity: Not reported						
10	Hussain et al. (2020)	Australia	391	Adults (aged 60 or over). Mild/Moderate ID) Mean age 65 (60–87)	Cross sectional survey: Use of a structured questionnaire.	Community	54%	Not available	Older age Female	Not available
	
				Males: 63% Females: 37%						
				Ethnicity: Not reported						
11	Cooper et al. (2015)	Scotland	8014	Adults (intellectual disability) Mean age: 43.1 (SD 15.8)	Cohort study: population based.	Primary care	41% (2 or more long term conditions)	Not available	Older age	Not available
				Male: 56% Female: 44%						
				Ethnicity: not reported						
12	Azimi et al. (2016)	Canada	78	Adults (intellectual disability) Mean age 31.6 (16–74)	Cohort study: based on case note review.	Psychiatric outpatients	97%	Not available	Not available	Not available
				Male: 69% Female: 31%						
				Ethnicity: Not reported						
13	McCarron et al. (2013)	Ireland	753	Adults (Intellectual disability, age 40 or over) Mean age 54.75 (SD 9.56)	Cohort study: Standardised protocol administered through face to face computer assisted interviews.	Community (data from the Irish longitudinal study on ageing (IDS-TILDA)	71%	Not available	Older age Female gender	Not available
				Males: 45% Females: 55%						
				Ethnicity: Not reported						
14	Van Timmeren et al. (2017)	Netherlands	99	Adults (severe or profound ID) Mean age 45.6 (SD 16.5)	Medical records review.	Residential care	Not available	Pairs of co-morbid conditions described	Not available	Not available
				Males: 50% Females: 50%						
				Ethnicity: Not reported						
15	Emerson et al. (2016)	UK	299	Adults (Intellectual disability) age range 16–49	Cohort study: Secondary analysis of data from a longitudinal study focusing on the life experiences of UK citizens).	Community sample	8% (2 or more conditions) 2% (3 or more conditions)	Not available	Not available	Not available
				Gender: Not reported						
				Ethnicity: Not reported						
16	Bayen et al. (2018)	USA	878	Adults (down syndrome; age 45 or over) Mean age 57 (45–89)	Cross-sectional study.	Community sample (using medicare claims database)	52% (2 or more conditions)	Co-morbidities in people with dementia and those without dementia described	Not available	Not available
				Males: 52% Females: 48%						
				White: 71% Hispanic: 20% Black: 5% Asian/Pacific: 3% Others: 2%						
17	Hermans and Evenhuis (2014)	Netherland	1047	Adults (50 years or over and Intellectual Disability) Mean age: 60.9 (50–65)	Cross sectional study.	Recruited from 3 large formal ID service providers (rural and urban)	80% (2 or more conditions) 47% (4 or more conditions)	Not described	Older age Severe or profound ID	Not available
				Male 51% Female 49%						
				Ethnicity: Not reported						
18	Kinnear et al. (2018)	Scotland	1023	Adults (Intellectual disability) Mean age 43.9 (16–83)	Cross-sectional study: Population-based	Community based	99% (2 or more conditions)	Not available	No association with neighbourhood deprivation. No difference between people with or without down syndrome	Not available
				Males: 55% Females: 45%						
				White: 96% Non-White: 4%						
19	Dunham et al. (2018)	Scotland	1023	Adults (intellectual disability) Mean age 43.9 (16–83)	Cross sectional study: Population based.	Community	94% (5 or more conditions)	Association between mental health and physical health conditions described	Not available	Not available
				Male: 55% Female: 45%						
				White: 96% Non-White: 4%						
20	Ptomey et al. (2020)	USA	13098 (adults 1383)	Children and adults (people who had down syndrome, autism or other intellectual and developmental disabilities)	Cohort study. Data extracted from an electronic database.	Data University Medical Center’s Healthcare Enterprise Repository	19% (2 or more conditions)	Not available	Not available	Not available
				Adults only Males: 40% Females: 60%						
				White: 78% Black: 13% Other: 9%						

### Study populations

The total number of individuals with intellectual disabilities from all of the 20 studies was 18,871. However, there was some overlap between studies that used the same cohort, so it was not possible to determine the actual number.

Most studies included only adults. Two studies (Emerson et al., 2016; Kinnear et al., 2018) included participants on or over the age of 16 although the majority included adults from the age of 18 only. Two further studies included children (Darbà, 2019; Ptomey et al., 2020). Six studies focused on ageing populations: one focused on ages 40+ years (*n* = 1, 5%), one on ages 45+ years (*n* = 1, 5%), three on ages 50+ years (*n* = 3, 15%), and one on ages 60+ years (*n* = 1, 5%). Only one study (*n* = 1, 5%) used both a lower and upper age limit; this focused on individuals aged 16–49 years (Emerson et al., 2016). All studies included both genders, but the gender distribution varied between studies with 40–86% males. Where reported, most individuals (71–96%) were white.

Studies included individuals with a wide range of intellectual disabilities. Almost half of the studies (*n* = 8, 40%) focused on chromosomal conditions or subgroups of people with intellectual disabilities/developmental disabilities: Down Syndrome (*n* = 3, 15%); autism spectrum disorders (ASD; *n* = 2, 10%); phenylketonuria (PKU; *n* = 1, 5%); 22q11.2 deletion syndrome (*n* = 1, 5%) and severe and multiple intellectual disabilities (*n* = 1, 5%). Just over half of the studies (*n* = 11, 55%) focused on people with defined severities of intellectual disability. Of these studies, one focused only on mild-moderate intellectual disability (Hussain et al., 2020) and one (Ptomey et al., 2020) included a further breakdown to include intellectual disability, ASD, Down Syndrome and any combinations of these.

### Study design and settings

A summary of the study design, population and settings are shown in Table 2. All studies were observational, using a range of approaches to identify and recruit individuals. The majority of studies (*n* = 12, 60%) evaluated data from publicly available health databases. The remainder of studies involved some current interaction with participants to measure health and compare this to wider data (*n* = 8, 40%). Studies were undertaken in a range of settings, including community independent living, supported placements, residential settings, and healthcare settings such as primary care and outpatient settings.

### Prevalence, nature and clusters of MLTC

The first two objectives for this scoping review involved determining the prevalence and nature of MLTC experienced by those with intellectual disabilities, and the assessment of whether some were more likely to occur together. For these objectives, we noted that the case mix of the study populations was generally not representative of individuals with intellectual disabilities as a whole. Almost half of all studies included in the review focused on individuals with one particular chromosomal syndrome or diagnosis thereby biasing towards MLTC experienced by people with these syndromes/diagnoses. For example, people with Down syndrome are more vulnerable to dementia than other people with intellectual disabilities (Sinai et al., 2018) and those with 22q11.2 deletion syndrome are vulnerable to congenital heart defects (Carotti et al., 2008).

### Prevalence and nature of MLTC

Table 2 summarises the prevalence of MLTC in each study. Most studies defined MLTC (“multimorbidity”) as two or more conditions, and one defined as four or more conditions.

The most common long-term conditions reported were epilepsy (*n* = 14, 70%), visual impairment (*n* = 10, 50%) and thyroid disorders (*n* = 9, 45%). Several conditions appeared in almost half of all studies (*n* = 8, 40%) including diabetes, hypertension, constipation/diarrhoea or mental health problems. Around one third (*n* = 7, 35%) reported hearing loss or skin conditions. A quarter of all studies (*n* = 5, 25%) reported on arthritis, dementia and obesity.

The prevalence of MLTC reported in the cohorts varied according to both the cohort size and characteristics, and list of conditions included in the definition of MLTC. The prevalence of MLTC, as defined by two or more conditions, varied from 8 to 99%. For three or more conditions, prevalence varied from 2 to 91%, and four or more conditions from 17 to 76%. Some reported the prevalence by age group, and found higher prevalence rates in older age groups. Papers generally reported the mean number of co-occurring health conditions, which ranged from 4 to 11.

Among all the studies included in the review and where comparisons were made with the general population, prevalence of MLTC was greater among people with intellectual disabilities than the general/majority population. Higher prevalence, higher number of conditions co-occurring and onset of multi-morbidity (presence of MLTC) much earlier in their lives were the key differences for population with ID.

### Clusters of MLTC

Although many of the studies reported long-term health conditions individually, only three studies (15%) included information on the prevalence of these conditions occurring together. Analysing a combined sample of people over 50 years with Down syndrome from two databases, Hodapp et al. (2019) described co-morbidities in people with dementia which included high cholesterol, diabetes, hypertension and other cardiovascular problems. The study by Tyrer et al. (2019) found that mental ill-health and obesity occurred most commonly together (15%), followed by mental ill-health and epilepsy (11%) and mental ill health and dysphagia (10%). Van Timmeren et al. (2017) reported on the most common combinations of physical health conditions, which were visual impairment and constipation (84%), followed by epilepsy and constipation (76%), and epilepsy and visual impairment (71%).

While this review identified high prevalence of MLTC in people with intellectual disabilities, the data from the literature about clusters or patterns of co-occurrence of these conditions in this population is limited as highlighted above. Where studies described co-occurrence, the description was limited to mostly commonly occurring pairs of conditions. Considering the higher number of multiple long-term conditions occurring together in this population, nature of this co-occurrences is a key knowledge.

### Risk factors for MLTC in patients with intellectual disabilities

The third objective of this review sought to identify risk factors for MLTC in adults with intellectual disabilities. Half of the papers (*n* = 10, 50%) offered no data in response to this objective. A review of the remaining literature identified the key risk factors: ageing; medication; obesity; specific conditions; demographics; and “other risk factors” such as exercise levels and non-participation in routine screening.

The most common risk factor reported in the literature (*n* = 7, 35%) was the link between ageing and MLTC. McCarron et al. (2013) found that those aged 65 or over were 3.4 times more likely to have MLTC than people aged 40–49 years. It is of note that, in this research, MLTC were high––even in the younger age groups (63% among those aged 40–49 years). Tyrer et al. (2019) reported that female gender was associated with increased prevalence of MLTC. McCarron et al. (2013) also found a large gender variation, reporting that females are almost twice as likely to have MLTC than males, regardless of age.

Belton et al. (2018) noted that people with intellectual disabilities as a whole were exposed to multiple medications (polypharmacy), some of which posed significant risks. Shoufour et al. (2018) also noted that, even after adjusting for MLTC, polypharmacy remained a strong independent predictor of mortality.

One paper referred to non-participation, or specifically a lack of experiencing a face-to-face appointment with a clinician, as a risk factor. Kinnear et al. (2018) reported that individuals with intellectual disabilities were significantly less likely to have a direct (face-to-face) encounter and significantly more likely have an indirect (i.e. not seen but clinical action taken) encounter.

### Outcome measures used to evaluate impact of MLTC

The final objective of this scoping review sought to identify outcome measures that could be used to evaluate the impact of MLTC in adults with intellectual disabilities. Although only one paper reviewed (*n* = 1, 5%) formally investigated outcomes in relation to MLTC, many of the reviewed articles discussed potential outcome measures that could be considered for future studies. These measures included mortality, cost of care, polypharmacy (use of multiple long term medications) and hospitalisations.

Polypharmacy could be considered as either a risk factor for MLTC or an outcome measure. Carfi et al. (2020) cited the link between ageing and polypharmacy as evidence of worsening existing conditions and Malecki et al. (2020) suggested use of number of medications as a proxy measure for comorbidities. Azimi et al. (2016) expressed concerns over side effects of psychotropic medications for people with MLTC.

Shoufour et al. (2018) collected mortality data and showed (for the first time) that MLTC and polypharmacy are strong predictors of mortality in people with intellectual disabilities, finding a 2.6-fold increased risk among those with MLTC compared to those without.

Overall, the findings of this review suggested that prevalence of MLTCs was much higher among people with intellectual disabilities and that epilepsy, sensory impairments, and mental ill-health were common co-occurring health conditions, particularly in older and obese individuals who were also vulnerable to polypharmacy. There was a distinct lack of evidence with regard to outcome measures or the impact of MLTCs in this population.

## Discussion

This scoping review adds significantly to what is known about MLTC in the intellectual disability population and identifies clear gaps for further work. We have a better insight into the long-term health conditions that people with intellectual disabilities experience, but still know very little about the co-occurrence of these conditions and whether some combinations of health conditions have worse outcomes than others. This is clearly an important area for future research as there is a need to develop future intervention strategies both to improve outcomes for these individuals and help to prevent these combinations of conditions from occurring in the first place.

Overall, the findings from this review highlight that long-term health conditions tend to occur earlier and at higher levels in patients with intellectual disabilities compared with the general population, which is consistent with evidence in the field (Bennett et al., 2021; Heslop and Hoghton, 2018; Mencap, 2021). The bulk of literature tends to focus on individual health conditions, rather than MLTC as evidenced in studies focusing on conditions, such as epilepsy (Bowley and Kerr, 2000; Kerr et al., 2009; Watkins et al., 2020), asthma (Davis, 2016; Gale et al., 2009; Xie et al., 2020), and diabetes (McVilly et al., 2014). However, the lack of data on MLTC and management of MLTC as a whole is a concern in terms of treating patients holistically and reducing polypharmacy.

Our review provided a small amount of evidence related to the risk factors for the intellectual disability population to develop MLTC. The findings suggest that age, gender, obesity and polypharmacy are potential risk factors. This is consistent with other work. Women are at greater risk than men (Reppermund et al., 2020) and risk increases with age (Hussain et al., 2020). Intellectual disability populations with obesity (Sadowsky et al., 2020) or polypharmacy use (Lim et al., 2021; McMahon et al., 2020) are at greater risk. Lack of face-to-face screenings or appointments have been raised as a concern in this population (Kinnear et al., 2018), which is an important consideration to take forward given the context of the pandemic which has become a threat and barrier to face to face healthcare services, in particular in the community (Flynn et al., 2021; Joy et al., 2020).

An important component of our review was to identify suitable outcome measures for studies of individuals with MLTC and intellectual disabilities. The limited studies available suggested that mortality, aggregated co-morbidity longitudinally and quality of life are important factors to consider as outcome measures for this population which is consistent with other recent work (Thurm et al., 2020). These outcomes are important to consider for future research as we found no literature to identify outcomes for combinations of long-term conditions. There is a clear need for further work to map co-occurrence of conditions to explore differential quality of life, morbidity and mortality outcomes. Strategies to prevent MLTC, including weight management (Ptomey et al., 2017; Patience, 2018) medication adherence (Resciniti et al., 2020) and early screening (Dunkley et al., 2017) are also important to prevent future complications.

This review has a number of key strengths and weaknesses. The limitations of this study are the relatively small number of papers included in the final review, although this seems to be mitigated by the overall number of people represented in the studies reviewed, and the consistency of this review with findings from a broader review of the literature for this discussion. The key strengths of the work are the consistency with findings across the field and reinforcement and refinement of some key and linked research questions related to the intellectual disabilities population. The gaps identified and recommendations made are a strength of this work as they act as a driver for further research in this important area which could have a significant impact on outcomes for this population.

Recommendations from this work include a need for further work to identify the co-occurrence of clusters of MLTC in people with intellectual disabilities. Although there are calls to treat MLTC together rather than in isolation (Suls et al., 2016; https://www.ncbi.nlm.nih.gov/pmc/articles/PMC4889007/), MLTC are typically combined into one problem. This hides the variation in complexity between individual long-term conditions, how they interact with each other, and any pharmacological-based interactions. Clusters of long-term conditions need to be mapped to a range of key outcome measures such as quality of life, morbidity and mortality to fill the current gap in data about which MLTC have the worst outcomes in people with intellectual disabilities. The potential risk factors identified in this study (gender, age, weight, medication use) should be considered in populations identified for future work, in order that they can be used in targeted interventions.

Secondly, we recommend specific guidance for people with intellectual disabilities as existing MLTC strategies are not always applicable to this population (NICE, 2016, 2020). For example, common long-term health conditions identified in this review, such as epilepsy and visual impairments, do not feature highly in general population research because they are relatively rare, so policy initiatives in the general population do not translate well to intellectual disability populations. Although there is recognition globally that MLTC are more common in disadvantaged groups, thereby contributing to health inequalities as a whole (World Health Organization, 2016), the lack of specific consideration of intellectual disabilities is, in our view, a concern. We know that people with intellectual disabilities have a high prevalence of MLTC but they may also lack communication skills, capacity to make certain decisions, and are known to be vulnerable to inequalities and inequities in health and healthcare provision (Heslop et al., 2013; Heslop and Hoghton, 2018). In most countries, general practitioners are the first point-of-contact for individuals with MLTC. In the UK, there are recommendations to increase appointment times both for people with intellectual disabilities (NHS England, 2021, Public Health England, 2016) and people with complex health conditions (British Medical Association, 2016). However, given limited resources, joined-up-care between health and social care services is likely to be equally important to ensure that the care needs of people with intellectual disabilities are adequately met.

### Implication for clinicians

This review indicates the need for better clinical awareness of the early onset of MLTC in people with intellectual disabilities. Many of these conditions are preventable and/or do not need to be life-limiting, if managed well (e.g. epilepsy; Beghi et al., 2015). Education of carers and clinicians, and better health screening to identify these conditions early and treat effectively is indicated. The strong relationship between the use of multiple long-term medications and MLTC indicate better vigilance of the potential effects of these medications and efforts to optimize medication use in this population.

## Conclusion

Overall, this review has highlighted that MLTC are a significant problem for people with intellectual disabilities and that there are significant gaps in the literature in relation to clusters of long-term conditions and how they affect mortality, future morbidities and quality of life. We call for more research in this area to identify prevention strategies and ways in which to improve outcomes for individuals affected. Finally, we recommend specific guidance for the care of people with intellectual disabilities and MLTC, which considers their complex physical, communication and behavioural needs.
